# Association between parenthood and cardiovascular disease risk: Analysis from NHANES 2011–2016

**DOI:** 10.1016/j.pmedr.2022.101820

**Published:** 2022-05-06

**Authors:** Cody D. Neshteruk, Katherine Norman, Sarah C. Armstrong, Rushina Cholera, Emily D'Agostino, Asheley C. Skinner

**Affiliations:** a Department of Population Health Sciences, Duke University Medical School, Durham, NC, United States; b Duke Clinical Research Institute, Duke University, Durham, NC, United States; c Department of Pediatrics, Duke University Medical School, Durham, NC, United States; d National Clinician Scholars Program, United States; e Duke Margolis Center for Health Policy, Duke University, Durham, NC, United States; f Department of Orthopaedic Surgery, Duke University Medical School, Durham, NC, United States

**Keywords:** Parents, Cardiovascular disease, Obesity, Physical activity, Family

## Abstract

The objective of this study was to examine the association between parenthood and cardiovascular disease (CVD) risk factors among a nationally representative sample of United States adults. A cross sectional analysis was conducted with adults aged 20–59 years from the National Health and Nutrition Examination Survey 2011–2016. Adults were classified as parents and non-parents based on the presence of children birth-17 years in the home. CVD risk factors assessed included: physical inactivity, obesity, blood pressure, HDL cholesterol, glycohemoglobin, and smoking status. Multivariable logistic regression models stratified by sex were used to examine the association between parenthood and each risk factor. 10,908 adults (5,329 [49%] male, weighted mean age 39.6 years) were included. In adjusted analyses, fathers had greater odds of obesity (OR: 1.22; 95% CI: 1.04–1.42) and lower odds of being a current smoker (OR: 0.82; 95% CI: 0.68–0.98) compared to non-fathers. Mothers had greater odds of physical inactivity (OR: 1.27; 95% CI: 1.03–1.56) and low HDL cholesterol (OR: 1.24; 95% CI: 1.06–1.45), and lower odds of being a current smoker (OR: 0.78; 95% CI: 0.63–0.96) compared to non-mothers. Parents with younger children in the household tended to have greater odds of CVD risk factors compared to non-parents. No clear patterns emerged in CVD factor risk based on the number of children in the household. Parents are at greater risk for several modifiable CVD risk factors. This illustrates the importance of including parental health promotion in settings that serve children and implementing policies that support parental health and wellbeing.

## 1 Introduction

Parenthood can be an emotionally rewarding experience, providing adults with a sense of purpose and meaning to their life. (Nelson et al., 2014) However, the physical, emotional, and financial demands of parenthood can contribute to decreased well-being and increased health risks in parents. (Umberson et al., 2010, Nomaguchi and Milkie, 2020) Compared to childless adults, parents tend to exhibit higher levels of stress and more frequently report depressive symptoms. (Hansen, 2012, Evenson and Simon, 2005) Parenthood is also associated with poorer lifestyle behaviors including declines in physical activity, increased sedentary behavior, less sleep and increased weight gain. (Bellows-Riecken and Rhodes, 2008, Berge et al., 2011, Garfield et al., 2016, Umberson et al., 2011, Corder et al., 2020, Abell et al., 2019, Carson et al., 2018) Parent health is especially influential on child health, as parents play a key role in supporting and promoting children’s healthy habits, serving as role models and structuring the environment in which children are raised. (Faith et al., 2012, Davison and Birch, 2001, Ventura and Birch, 2008) Furthermore, worse parent health could contribute to difficulties in caring for children, limit the time parents can spend with their children and the types of activities they can do together, and promote financial instability through missed work and increased healthcare costs. (Pollmann-Schult, 2014) Better understanding the health impacts associated with parenthood could provide important evidence for tailoring preventative services to the needs of parents as well as addressing parent health behaviors as part of pediatric care.

Cardiovascular disease is the leading cause of adult mortality in the United States (US) and it is well established that the presence of cardiovascular disease risk factors are evident as early as childhood. (Kavey et al., 2003) Studies measuring cardiovascular disease risk factors in children have shown associations between similar risk factors in their parents, indicating that risk factors may cluster at the family level. (Khoury et al., 2016, Reis et al., 2006, Schwandt et al., 2009) Despite these findings, there remains a dearth of information about the cardiovascular disease risk of parents, particularly within the broader US population. Therefore, the aim of this study was to examine the association between parenthood and cardiovascular disease risk factors among a nationally representative sample of US adults.

## 2 Methods

*Study population.* This study uses National Health and Nutrition Examination Survey (NHANES) data from 2011 to 2016. NHANES is a stratified, multistage probability sample of the noninstitutionalized US population. It includes an in-home interview, physical examination, and laboratory measurements. A detailed description of NHANES is available elsewhere. (Health and Survey, 2020) Participants who were 20–59 years at the time of examination (n = 11,515) were included. Pregnant women (n = 192) and participants without sample weights for the physical examination (n = 415) were excluded, yielding 10,908 participants included in the current analysis. This analysis used only de-identified secondary data and was deemed exempt from further review by the Duke University Institutional Review Board under federal regulation 45 CFR §46.101(b).

*Parent status.* Beginning in the 2011–2012 cycle, during the in-home interview, participants responded to two items assessing the number of children living in the household. The first item captured the total number of children 0–5 years living in the household and the second captured the total number of children 6–17 years in the household. The presence of children in the household (yes/no) was used to define parents and non-parents. While, we are not able to determine if adults are parents, other relatives, or unrelated, for most households, the adults are likely caregivers of the children, regardless of parenthood. We refer to adults with children residing in their home as “parents” for simplicity. Secondary exposure variables included the age of the youngest child in the household (none, 0–5, and 6–17) and the total number of children in the home (0, 1, 2, and 3 or more).

*Cardiovascular disease risk factors.* Cardiovascular disease risk factors assessed included physical inactivity, obesity, high blood pressure, elevated glycated hemoglobin, low HDL cholesterol, and current smoking status.

Self-reported leisure time physical activity was assessed during the in-home interview. Participants were asked if they engaged in moderate and vigorous intensity sports, fitness or recreation activities during a typical week (yes/no). If they responded yes, follow-up questions assessed the typical number of days per week and minutes per day for each intensity. Total minutes per week of leisure time physical activity were calculated as the minutes of moderate physical activity plus two times the minutes of vigorous physical activity. The Physical Activity Guidelines for Americans recommend that adults obtain at least 150 min of moderate aerobic physical activity per week, 75 min of vigorous physical activity per week, or an equivalent combination, thus vigorous physical activity was doubled to account for the higher intensity. (Piercy et al., 2018) Participants were then categorized as active or inactive based on meeting the national recommendation of at least 150 min of moderate or vigorous intensity aerobic activity per week.

Height and weight were collected during the physical examination using standardized procedures and used to calculate body mass index (BMI, kg/m^2^). Participants with a BMI ≥ 30 kg/m^2^ were classified as having obesity.

Blood pressure was measured during the physical examination by a trained physician. Once the participant was sitting quietly for five minutes, three consecutive blood pressure measures were obtained. If one of the measures was interrupted or incomplete, a fourth measure could be taken. The average of all blood pressure measurements was calculated. Participants with either systolic blood pressure ≥ 130 mm Hg or with diastolic blood pressure ≥ 80 mm Hg, or who indicated they were taking medication for hypertension, were classified as having high blood pressure. (Carey and Whelton, 2018).

Glycated hemoglobin levels, HDL cholesterol, and smoking status were assessed as part of the laboratory measurement. A detailed description of the collection and measurement procedures can be found in the NHANES Laboratory Procedures Manual. (National Health and Nutrition Examination Survesy, 2017) A glycated hemoglobin value ≥ 5.7% was defined as elevated risk for diabetes based on recommendations from the American Diabetes Association. (Classification and Diagnosis of Diabetes, 2020) HDL cholesterol values < 40 mg/dL for men and < 50 mg/dL for women were classified as low HDL cholesterol based on current recommendations. (Grundy et al., 2019) Smoking status was determined by measuring serum cotinine, a biomarker of nicotine. Smokers were defined as having a cotinine value ≥ 3.08 ng/mL based on prior analyses with the NHANES dataset. (Benowitz et al., 2009).

*Covariates.* Information on participant age, race/ethnicity, marital status, and income were collected. Race and ethnicity were categorized as non-Hispanic white, non-Hispanic black, Hispanic or Mexican American, and other. Marital status was dichotomized as married or living with partner and not married or living with partner. Income was based on percent of the federal poverty level and included the following categories < 200% (most deprived), 200–399%, and ≥ 400 (most affluent). Because of a large amount of missing data for income, a missing category was also included to maximize the sample size.

*Analysis.* Bivariate analyses were conducted examining the prevalence of cardiovascular disease risk factors by parent status and sex. All comparisons were tested using adjusted Wald tests. Only the test of difference across all categories is provided along with 95% confidence intervals. Multivariable logistic regression models were used to examine the association between parent status and each cardiovascular disease risk factor stratified by sex. Additional models examined the association between age of the youngest child, number of children, and cardiovascular disease risk factors. All models were adjusted for participant age, race/ethnicity, marital status, income and NHANES cycle and used non-parents as the reference group. All analyses were adjusted to account for the complex NHANES survey design and were performed using PROC SURVEY in SAS 9.4 (SAS, Cary, North Carolina).

## 3 Results

The sample (n = 10,908) included 5,329 males (48.8%) and 5,579 females (51.2%). Table 1 reports the demographic characteristics of the full sample stratified by participant sex and is representative of the US population of adults 20–59 years old.

**Table 1:** Demographic characteristics by sex of adults 20–59 years from NHANES 2011–2016 (n = 10908).

**Characteristics**	**No (%) of participants**^a^	
	**Men** (n = 5329)	**Women** (n = 5579)
Age, years		
20–29	1377 (26.0)	1314 (24.1)
30–39	1372 (24.0)	1377 (23.5)
40–49	1282 (24.6)	1503 (26.0)
50–59	1298 (25.5)	1385 (26.3)
Race/ethnicity		
Non-Hispanic white	1912 (61.9)	1885 (61.0)
Non-Hispanic black	1166 (11.4)	1306 (13.1)
Hispanic/Mexican American	1268 (17.8)	1426 (16.7)
Other	983 (9.0)	962 (9.2)
Marital status ^b^		
Not married	2053 (37.3)	2358 (38.3)
Married or living with partner	3274 (62.7)	3221(61.7)
Percent of federal poverty level		
< 200%	2354 (34.0)	2587 (36.4)
200–399%	1241 (25.4)	1298 (25.6)
≥ 400%	1294 (34.0)	1264 (31.9)
Missing	440 (6.6)	430 (6.2)
Children in the home		
No	2645 (53.9)	2325 (48.4)
Yes	2684 (46.1)	3254 (51.6)
Number of children		
0	2645 (53.9)	2325 (48.4)
1	1004 (18.0)	1207 (20.2)
2	914 (16.6)	1117 (18.6)
3 or more	766 (11.5)	930 (12.8)
Age of youngest child, years		
None	2645 (53.9)	2325 (48.4)
0 to 5	1349 (21.8)	1616 (24.4)
6 to 17	1335 (24.2)	1638 (27.2)
Abbreviation: National Health and Nutrition Examination Survey (NHANES)(a) Sample size and weighted frequency for United States population(b) Missing data: marital status (n = 2)		

*Prevalence of cardiovascular disease risk factors.* Unadjusted analyses (Table 2) show that among women, mothers compared to non-mothers had a higher prevalence of physical inactivity (57.0% vs. 63.9%), obesity (37.1% vs. 40.6%), and low HDL cholesterol (27.1% vs. 36.1%). Compared to non-mothers, mothers also had a lower prevalence of high blood pressure (36.7% vs. 28.2%). Among men, fathers compared to non-fathers had a higher prevalence of obesity (32.7% vs. 38.1%) and low HDL cholesterol (25.3% vs. 31.1%). Compared to non-fathers, fathers also had a lower prevalence of high blood pressure (43.7% vs. 39.1%) and smoking (35.7% vs. 30.8%).

**Table 2:** Unadjusted prevalence of cardiovascular disease risk factors by sex and parent status for adults 20–59 years from NHANES 2011–2016 ^a,b.^

**Characteristics**	**Men**								**Women**						
	Without children (n = 2644)			With children (n = 2683)					Without children (n = 2325)			With children (n = 3254)			
	n	Prevalence (95% CI)		n	Prevalence (95% CI)		p value		n	Prevalence (95% CI)		n	Prevalence (95% CI)		p value
Physical inactivity															
No	1198	46.2 (43.1–49.4)		1109	43.3 (40.8–45.7)		0.13		878	43.0 (39.2–46.8)		1063	36.1 (33.8–38.4)		<0.001
Yes	1447	53.8 (50.7–56.9)		1575	56.7 (54.3–59.2)				1447	57.0 (53.2–60.8)		2191	63.9 (61.6–66.2)		
Obesity															
No	1803	66.6 (63.7–69.6)		1691	60.9 (58.1–63.7)		<0.01		1363	62.3 (59.6–65.0)		1842	58.5 (56.4–60.6)		0.03
Yes	824	32.7 (29.7–35.7)		966	38.1 (35.5–40.7)				939	37.1 (34.4–39.8)		1378	40.6 (38.6–42.6)		
High blood pressure															
No	1428	54.4 (51.0–57.7)		1500	58.0 (55.2–60.9)		0.04		1364	61.2 (58.2–64.2)		2132	67.5 (64.7–70.2)		<0.001
Yes	1165	43.7 (40.2–47.2)		1089	39.1 (36.5–41.8)				913	36.7 (33.5–39.9)		969	28.2 (25.8–30.5)		
Elevated glycated hemoglobin															
No	1759	72.1 (69.5–74.8)		1767	73.2 (70.6–75.8)		0.68		1518	72.1 (69.3–74.9)		2273	74.1 (72.1–76.1)		0.26
Yes	760	24.0 (21.7–26.3)		779	22.7 (20.3–25.1)				713	24.5 (21.9–27.1)		838	22.0 (20.2–23.9)		
Low HDL cholesterol															
No	1875	70.2 (67.7–72.7)		1693	64.3 (62.0–66.7)		<0.01		1494	67.9 (65.3–70.6)		1856	59.4 (57.0–61.9)		<0.001
Yes	619	25.3 (22.9–27.8)		835	31.1 (29.0–33.3)				701	27.1 (24.7–29.5)		1226	36.1 (33.4–38.9)		
Current smoker															
No	1503	59.8 (56.3–63.3)		1656	64.8 (62.3–67.3)		0.02		1656	71.0 (68.1–73.9)		2396	74.0 (71.9–76.0)		0.24
Yes	991	35.7 (32.3–39.1)		877	30.8 (28.6–33.1)				544	24.2 (21.4–27.1)		691	21.8 (19.9–23.6)		
Abbreviation: National Health and Nutrition Examination Survey (NHANES) Sample size and weighted frequency for United States population Not all prevalence frequencies will add up to 100% because of missing data															

*Parent status.* In adjusted analyses, fathers had greater odds of obesity (OR: 1.22; 95% CI: 1.04–1.42) and lower odds of being a current smoker (OR: 0.82; 95% CI: 0.68–0.98) compared to non-fathers (Table 3**)**. Mothers had greater odds of physical inactivity (OR: 1.27; 95% CI: 1.03–1.56) and low HDL cholesterol (OR: 1.24; 95% CI: 1.06–1.45) and lower odds of being a current smoker (OR: 0.78; 95% CI: 0.63–0.96) compared to non-mothers.

**Table 3:** Adjusted odds of cardiovascular disease risk factors by sex and parent status for adults 20–59 years old from NHANES 2011–2016 ^a.^

	**Physical inactivity** (n = 10906)		**Obesity** (n = 10805)		**High blood pressure** (n = 10558)		**Elevated glycated hemoglobin** **(**n = 10405)		**Low HDL cholesterol** (n = 10297)		**Current smoker** (n = 10312)	
	OR (95% CI)	p	OR (95% CI)	p	OR (95% CI)	p	OR (95% CI)	p	OR (95% CI)	p	OR (95% CI)	p
**Children in home**												
Men												
No	Ref		Ref		Ref		Ref		Ref		Ref	
Yes	1.05 (0.90–1.22)	0.56	1.22 (1.04–1.42)	0.02	0.99 (0.82–1.19)	0.87	0.91 (0.72–1.15)	0.40	1.18 (1.00–1.40)	0.05	0.82 (0.68–0.98)	0.03
Women												
No	Ref		Ref		Ref		Ref		Ref		Ref	
Yes	1.27 (1.03–1.56)	0.03	1.07 (0.92–1.24)	0.36	0.90 (0.73–1.12)	0.34	1.00 (0.81–1.25)	0.97	1.24 (1.06–1.45)	<0.01	0.78 (0.63–0.96)	0.02
Abbreviations: National Health and Nutrition Examination Survey (NHANES); odds ratio (OR); confidence interval (CI)(a) Models adjusted for: age, race/ethnicity, marital status, income, and NHANES cycle												

*Age of the youngest child.* Only fathers of younger children had greater odds of physical inactivity (OR: 1.21; 95% CI: 1.01–1.45), obesity (OR: 1.24; 95% CI: 1.06–1.45), and low HDL cholesterol (OR: 1.31; 95% CI: 1.07–1.61) (Table 4). For mothers, only those with younger children had increased odds of physical inactivity (OR: 1.47; 95% CI: 1.17–1.85). Mothers of younger children also had a lower odds of high blood pressure (OR: 0.76, 95% CI: 0.60–0.97) and being a current smoker (OR: 0.74; 95% CI: 0.61–0.90).

**Table 4:** Adjusted odds of cardiovascular disease risk factors by sex, age of the youngest child and number of children for adults 20–59 years old from NHANES 2011–2016^a^.

	**Physical inactivity**		**Obesity**		**High blood pressure**		**Elevated glycated hemoglobin**		**Low HDL cholesterol**		**Current smoker**	
	OR (95% CI)	p	OR (95% CI)	p	OR (95% CI)	p	OR (95% CI)	p	OR (95% CI)	p	OR (95% CI)	p
**Age of child**												
Men												
No child	Ref		Ref		Ref		Ref		Ref		Ref	
0–5 years	1.21 (1.01–1.45)	0.04	1.24 (1.06–1.45)	<0.01	1.06 (0.87–1.30)	0.57	0.95 (0.72–1.26)	0.71	1.31 (1.07–1.61)	0.01	0.89 (0.72–1.09)	0.24
6–17 years	0.94 (0.78–1.13)	0.51	1.20 (0.98–1.47)	0.08	0.94 (0.76–1.16)	0.54	0.88 (0.68–1.14)	0.32	1.10 (0.91–1.33)	0.34	0.77 (0.62–0.95)	0.01
Women												
No child	Ref		Ref		Ref		Ref		Ref		Ref	
0–5 years	1.47 (1.17–1.85)	<0.01	0.99 (0.84–1.17)	0.92	0.76 (0.60–0.97)	0.03	0.94 (0.74–1.19)	0.58	1.17 (0.97–1.42)	0.10	0.74 (0.61–0.90)	<0.01
6–17 years	1.13 (0.90–1.41)	0.29	1.13 (0.96–1.34)	0.15	0.99 (0.77–1.25)	0.90	1.04 (0.81–1.34)	0.74	1.30 (1.09–1.54)	<0.01	0.80 (0.62–1.05)	0.10
**Number of children**												
Men												
0	Ref		Ref		Ref		Ref		Ref		Ref	
1	0.99 (0.81–1.21)	0.94	1.30 (1.08–1.56)	<0.01	1.01 (0.82–1.25)	0.91	1.01 (0.76–1.33)	0.95	1.30 (1.03–1.63)	0.03	0.87 (0.69–1.10)	0.24
2	0.98 (0.79–1.22)	0.85	1.07 (0.84–1.36)	0.57	0.90 (0.71–1.14)	0.36	0.78 (0.59–1.04)	0.09	1.00 (0.78–1.28)	0.99	0.81 (0.64–1.02)	0.07
3 or more	1.32 (1.04–1.68)	0.02	1.30 (1.04–1.62)	0.02	1.09 (0.81–1.48)	0.57	0.91 (0.67–1.23)	0.54	1.25 (0.97–1.60)	0.08	0.72 (0.52–0.99)	0.04
Women												
0	Ref		Ref		Ref		Ref		Ref		Ref	
1	1.29 (0.98–1.70)	0.07	1.19 (1.02–1.40)	0.03	1.03 (0.79–1.34)	0.81	1.16 (0.91–1.49)	0.22	1.30 (1.07–1.57)	<0.01	0.95 (0.70–1.28)	0.72
2	1.34 (1.08–1.67)	0.01	0.95 (0.79–1.14)	0.59	0.79 (0.60–1.04)	0.09	0.98 (0.73–1.31)	0.88	1.18 (0.95–1.46)	0.14	0.63 (0.49–0.80)	<0.001
3 or more	1.09 (0.84–1.42)	0.50	1.01 (0.79–1.29)	0.95	0.82 (0.62–1.09)	0.16	0.71 (0.50–1.01)	0.05	1.22 (0.96–1.55)	0.11	0.68 (0.52–0.90)	<0.01
Abbreviations: National Health and Nutrition Examination Survey (NHANES); odds ratio (OR); confidence interval (CI)(a) Models adjusted for: age, race/ethnicity, marital status, income, and NHANES cycle												

*Number of children.* No clear pattern of cardiovascular risk factors emerged based on the number of children in the household (Table 4). Fathers with three or more children had greater odds of physical inactivity (OR: 1.32; 95% CI: 1.04–1.68) and obesity (OR: 1.30; 95% CI: 1.04–1.62) and lower odds of being a current smoker (OR: 0.72; 95% CI: 0.52–0.99) compared to non-fathers (Table 4). Fathers with one child had greater odds of obesity (OR: 1.30; 95% CI: 1.08–1.56) and low HDL cholesterol (OR: 1.30; 95% CI: 1.03–1.63) compared to non-fathers. Similarly, mothers with one child had greater odds of obesity (OR: 1.19; 95% CI: 1.02–1.40) and low HDL cholesterol (OR: 1.30; 95% CI: 1.07–1.57) compared to non-mothers. Mothers with two children had greater odds of physical inactivity (OR: 1.34; 95% CI: 1.08–1.67) and lower odds of being a current smoker (OR: 0.63; 95% CI: 0.49–0.80) compared to non-mothers.

## 4 Discussion

Little is known about how parenthood is associated with cardiovascular disease risk in the US. To address this gap, we used nationally representative survey data from NHANES to examine the association between parenthood and key cardiovascular disease risk factors in adults 20–59 years old. Our findings reinforce prior evidence that parenthood is associated with lower physical activity and obesity. We also found that parents had greater odds of low HDL cholesterol and lower odds of being current smokers. Associations were generally similar among men and women, by age of the youngest child, and by the number of children in the household. These findings illustrate the importance of developing practices and policies that support parental health and wellbeing.

Parents had increased odds of several cardiovascular disease risk factors in this study. Consistent with prior research, parents tend to be less active and experience increased weight status. (Bellows-Riecken and Rhodes, 2008, Umberson et al., 2011) The responsibilities of child rearing likely limits the amount of time and opportunities for physical activity, which combined with poorer dietary behaviors, contributes to increased weight status in parents. (Mailey et al., 2014, Nezami et al., 2020, Welch et al., 2009) Furthermore, young and middle adulthood is a period of accelerated weight gain, which may be exacerbated by parenthood. (Dutton et al., 2016) A novel finding from this study was that parents had greater odds of having low HDL cholesterol. This may be attributed in part to our findings around physical activity and obesity, key drivers of low HDL cholesterol. Although not examined in this study, dietary intake may be different among parents and non-parents, which could also influence HDL cholesterol. (Nasuti et al., 2014) Future studies are needed to investigate the association between dietary intake and cardiovascular disease risk in parents.

Both mothers and fathers in this study had lower odds of being current smokers. Public health efforts have made it a priority to reduce children’s exposure to tobacco smoke and parental smoking is the primary source of tobacco smoke exposure for children. (Organization, 2017, Matt et al., 2004) It may be that the decision to have children motivates adults to quit smoking, particularly mothers because of the known risk of smoking during pregnancy. While these findings are promising, effective strategies to reduce parent smoking are still needed. A recent systematic review and *meta*-analysis showed that smoking cessation interventions delivered by pediatric health care professionals had little effect on parent smoking. (Daly et al., 2016).

Our findings suggest that early years of parenthood may be a particularly important time in helping parents manage their health and health behaviors. When examining age of the youngest child, parents with younger children tended to have greater odds of cardiovascular disease risk factors. During this time, children require the greatest amount of care and parents likely sacrifice their own health to care for their children. Fathers of young children in particular had increased odds of multiple risk factors relative to non-fathers and fathers with older children, indicating that fathers of young children may be an especially vulnerable population. Fathers tend to be less involved in children’s healthcare, but when they are, children experience more positive outcomes. (Yogman et al., 2016) Future studies should consider how to engage fathers in family-based health promotion in the clinical setting in order to benefit both fathers and children.

We found no clear patterns in the association between parenthood and cardiovascular disease risk based on the number of children. Based on previous studies, we hypothesized that the more children in the home, the greater the cardiovascular risk, as more children may signify added stress and greater economic burden. (Lawlor et al., 2003, Parikh et al., 2010) Additionally, some studies have suggested that pregnancy may increase cardiovascular disease risk in women, but this study and several others show that there are similar risks among both women and men with children, indicating that lifestyle factors associated with parenthood may be particularly important in modifying parent cardiovascular disease risk. (Hardy et al., 2009, Hardy et al., 2007).

While family-based interventions are acknowledged as important strategies for children’s health promotion and the prevention and treatment of chronic diseases, gaps remain in supporting parents to engage in healthy lifestyle behaviors. As an example, while child obesity interventions tend to be most effective when parents are included, the focus remains on helping the child, rather than helping parents themselves be healthier. (Mehdizadeh et al., 2020) Findings from our study suggest that family-based approaches in pediatric care, especially those around obesity and cardiovascular health, can use these opportunities to simultaneously target parental health behaviors, rather than just improving child health. For instance, untreated postpartum depression can adversely affect the well-being of children and families. As a result, the American Academy of Pediatrics recommends that pediatric clinics screen for postpartum depression at well-child visits throughout infancy and provide treatment resources and referrals for parents who screen positive. (Earls et al., 2019).

Policy interventions have enormous potential to improve the health and wellbeing of parents and directly impact child health. For example, universal paid family and medical leave (PFML) policies have well-documented benefits in improving the mental and physical health of mothers and children, breastfeeding duration, paternal engagement and care-taking, and positive population-level socioeconomic effects. (Van Niel et al., 2020, Petts et al., 2020) Despite these benefits, the U.S. remains the only industrialized nation to lack a national paid maternity leave policy and currently only eight states have comprehensive PFML legislation in place. (Montez et al., 2020) Expanding Medicaid eligibility to low-income parents is another policy intervention with clear positive impact on parental access to care, parental psychological stress, receipt of recommended care for both parents and children, and insurance coverage for children.(McMorrow et al., 2017, Lipton, 2021, Hudson and Moriya, 2017) Such family-friendly policy interventions can improve the health of parents, though further studies should examine the specific impact of policies on cardiovascular risk factors.

Strengths of this study included the use of a large, nationally representative sample of US adults and the use of objective measures to define cardiovascular disease risk. However, there were several limitations. First, with the use of a cross-sectional design, we cannot infer causation or understand how cardiovascular risk may change over time. Future studies should consider examining the cardiovascular disease risk of parents using prospective cohort or randomized controlled trial designs to assess the effects of parenthood on cardiovascular disease risk. Second, we made the assumption that participants were parents based on the presence of children in the house. There is no variable available in the NHANES dataset to assess parent status. It is likely that not all individuals living with children are parents. However, this may capture a variety of family structures as well as represent the health risks of being a caregiver, not just a parent. Additionally, while we looked at differences among mothers and fathers, future studies should consider examining differences in cardiovascular disease risk among parents by other factors including demographics (e.g., race/ethnicity) and environmental (e.g., neighborhood built environment) characteristics in order to further identify specific subgroups for intervention. Finally, we did not include diet as a cardiovascular disease risk factor. This may help explain some of our findings, particularly around obesity and HDL cholesterol. Future studies should explore how dietary patterns differ among parents and non-parents.

## 5 Conclusion

Adverse parent health can make caring for children difficult, provide additional strain on parents, and promote unhealthy behaviors in children. Findings from this study show that parents have greater odds of several cardiovascular disease risk factors that are likely attributed to modifiable lifestyle factors. Using approaches to caring for children that also emphasize the health of parents, can help pediatric providers improve the health and wellbeing of both parents and their children.

## CRediT authorship contribution statement

**Cody D. Neshteruk:** Conceptualization, Formal analysis, Methodology, Writing – original draft, Writing – review & editing. **Katherine Norman:** Formal analysis, Writing – review & editing. **Sarah C. Armstrong:** Conceptualization, Methodology, Writing – original draft, Writing – review & editing. **Rushina Cholera:** Writing – review & editing. **Emily D'Agostino:** Writing – review & editing. **Asheley C. Skinner:** Conceptualization, Methodology, Writing – original draft, Writing – review & editing.

## Declaration of Competing Interest

The authors declare that they have no known competing financial interests or personal relationships that could have appeared to influence the work reported in this paper.
